# Increased Risk of Active Tuberculosis following Acute Kidney Injury: A Nationwide, Population-Based Study

**DOI:** 10.1371/journal.pone.0069556

**Published:** 2013-07-25

**Authors:** Vin-Cent Wu, Chen-Yi Wang, Chih-Chung Shiao, Chia-Hsui Chang, Hui-Yu Huang, Tao-Min Huang, Chun-Fu Lai, Meng-Chun Lin, Wen-Je Ko, Kwan-Dun Wu, Chong-Jen Yu, Chin-Chung Shu, Chih-Hsin Lee, Jann-Yuan Wang, the National Taiwan University Study Group on Acute Renal Failure (NSARF), Taiwan Anti-Mycobacteria Investigation (TAMI) Group

**Affiliations:** 1 Department of Internal Medicine, National Taiwan University Hospital, Taipei, Taiwan; 2 Department of Internal Medicine, and Medical Research Center, Cardinal Tien Hospital, Fu Jen Catholic University, Xindian Dist, New Taipei City, Taiwan; 3 Division of Nephrology, Department of Internal Medicine, Saint Mary's Hospital, and Saint Mary's Medicine, Nursing, and Management College, Luodong, Yilan; 4 Yun-Lin Branch, National Taiwan University Hospital, Taipei, Taiwan; 5 Department of Traumatology, National Taiwan University Hospital, Taipei, Taiwan; 6 NSARF, The National Taiwan University Study Group on Acute Renal Failure, Taipei, Taiwan; 7 TAMI, Taiwan Anti-Mycobacteria Investigation Group, Taipei, Taiwan; Beijing Institute of Microbiology and Epidemiology, China

## Abstract

**Background:**

Profound alterations in immune responses associated with uremia and exacerbated by dialysis increase the risk of active tuberculosis (TB). Evidence of the long-term risk and outcome of active TB after acute kidney injury (AKI) is limited.

**Methods:**

This population-based-cohort study used claim records retrieved from the Taiwan National Health Insurance database. We retrieved records of all hospitalized patients, more than 18 years, who underwent dialysis for acute kidney injury (AKI) during 1999–2008 and validated using the NSARF data. Time-dependent Cox proportional hazards model to adjust for the ongoing effect of end-stage renal disease (ESRD) was conducted to predict long-term de novo active TB after discharge from index hospitalization.

**Results:**

Out of 2,909 AKI dialysis patients surviving 90 days after index discharge, 686 did not require dialysis after hospital discharge. The control group included 11,636 hospital patients without AKI, dialysis, or history of TB. The relative risk of active TB in AKI dialysis patients, relative to the general population, after a mean follow-up period of 3.6 years was 7.71. Patients who did (hazard ratio [HR], 3.84; *p*<0.001) and did not (HR, 6.39; *p*<0.001) recover from AKI requiring dialysis had significantly higher incidence of TB than patients without AKI. The external validated data also showed nonrecovery subgroup (HR = 4.37; p = 0.049) had high risk of developing active TB compared with non-AKI. Additionally, active TB was associated with long-term all-cause mortality after AKI requiring dialysis (HR, 1.34; *p* = 0.032).

**Conclusions:**

AKI requiring dialysis seems to independently increase the long-term risk of active TB, even among those who weaned from dialysis at discharge. These results raise concerns that the increasing global burden of AKI will in turn increase the incidence of active TB.

## Introduction

Tuberculosis (TB) accounts for a significant proportion of all deaths caused by infectious diseases. Controlling TB is a major public health issue, especially in developing countries. The relative risk (RR) of developing active TB is 10–25times for patients with chronic kidney disease (CKD) or those on hemodialysis, and 37times for renal transplant recipients than general population [1], and their TB mortality rate is higher [2], [3]. The incidence of dialysis –requiring AKI in the United States is now higher than the incidence of end-stage renal disease (ESRD), averaging a 10% increased per year [4] and is associated with increased use of resources during and after hospitalization [5]. Depending on how AKI is defined, 7–18% of all hospitalized patients suffer from AKI [6], and as many as 5% of intensive care patients have AKI severe enough to require dialysis [7]. More hospitalized patients with AKI are discharged after temporary AKI [8], perhaps because of advances in critical care medicine and dialysis technologies.

Profound alterations in immune responses associated with uremia and exacerbated by dialysis increase the risk of active TB [9]. Some individuals, especially those with impaired T cell function, develop active tuberculosis, either as primary progression or as a reactivation [10]. Kidney disease is associated with acquired immunodeficiency due to functional abnormalities of neutrophils, reduced T cell and B cell function, and compromised monocyte and natural killer cell function [11]. Increased proinflammatory cytokines, leukocyte trafficking directly mediate pulmonary injury after AKI [12]. Evidence of the long-term risk and outcome of active TB after advanced AKI is limited.

The current study used a population-based and record-based case-control design to evaluate the association between AKI requiring dialysis and long-term risk of active TB using the National Health Insurance Research Database of Taiwan.

## Methods

### Study sample

The Taiwan National Health Insurance (NHI) database is a nationwide insurance program covering outpatient visits, hospital admissions, prescriptions, intervention procedures, and disease profiles for over 99% of the population in Taiwan (23.12 million in 2009) [13], [14], [15]. It is one of the largest and most comprehensive databases in the world and has been used extensively in various studies of prescription use, diagnoses, and hospitalizations.

In cooperation with the Bureau of NHI, the NHRI of Taiwan used a systematic, random sampling method to build a representative database of 1,000,000 NHI enrollees [13], [14], [16] . There were no statistically significant differences in age, sex, or health-care costs between the sample group and all the enrollees, as reported by the NHRI. This data set spans from January 1, 1999, through December 2008 and includes all claims data for these 1,000,000 individuals. It offers a good opportunity to explore outcomes of patients with AKI requiring dialysis.

### Ethics Statement

*Because the identification numbers of all subjects in the NHRI databases were encrypted to protect the privacy of the individuals, this study was exempted from full review by the Institution Review Board of the National Taiwan University Hospital and informed consents were waived.*

### Population-based surveillance methods

**Figure 1:**
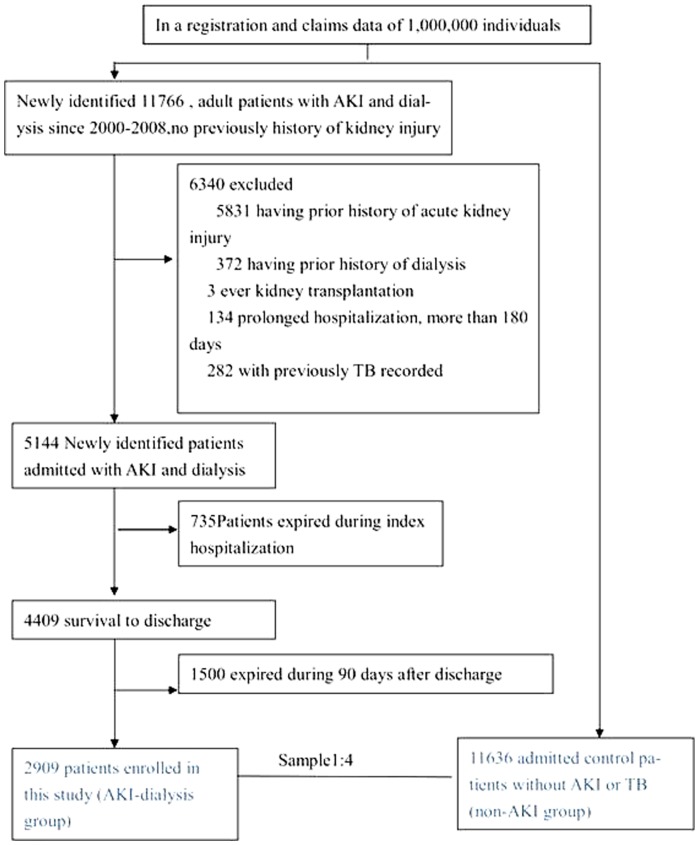
Flow diagram of the study sample. (Abbreviations; AKI, acute kidney injury; CKD, chronic kidney disease; TB, tuberculosis; ESRD, end-stage renal disease; ICU, intensive care unit).

All patients, aged >18 years, and who had a first-time admission diagnosis of AKI requiring dialysis were identified. Those who survived for >90 days after discharge without re-hospitalization (AKI–dialysis group) were selected for further analysis. Patients were analyzed from January 2000 through December 2008 to confirm that there was no diagnosis of AKI, dialysis, or TB within 1 year prior to index admission. Comorbidities were defined according to the International Classification of Disease, 9^th^ revision, Clinical Modification (ICD-9-CM) and procedure codes (including Taiwan Classification of Procedures). We excluded patients requiring prolonged hospitalization and those who underwent kidney transplantation. Figure 1 depicts the NHRI procedure for building the AKI database and selecting patients. A control cohort, 11636 patients without AKI, dialysis, or TB before and during non-AKI hospitalization (non-AKI group), was randomly selected. Four controls were selected for each AKI–dialysis case. However, we could not identify different causes of AKI from this retrospective database.

Patients in the AKI–dialysis group were further divided into 2 sub-groups, based on whether they continued dialysis (recovery and nonrecovery sub-groups). As in our previously report [17], we used a selection period of 90 days [18] to reduce the possibility of immortal time bias [19], because all patients after dialysis for more than 90 days in Taiwan will apply to the NHI for catastrophic illness registration cards.

### Research variables

Charlson comorbidity index scores were based on pre-existing conditions identified from a patient's medical records [20].

Demographic and clinical characteristics of study subjects and hospitals at index hospital admission were examined, including age, sex, year of admission, and prevalence of selected comorbidities. Data representing the hospitalization period included diagnosis codes; the categories of major operations; resource use, including hemodialysis; mechanical ventilation (MV); intensive care unit (ICU) admission; and outcomes. To determine pre-existing comorbidities, we used a relatively strict criterion: at least 1 admission or at least 3 outpatient visits for treatment of a certain disease during the year prior to the index admission.

### Definition of outcomes

The government of Taiwan monitors active TB, and anti-TB drugs are controlled by a central government health institute. Doctors must report every new case to the Taiwan center of disease control (CDC) within 1 week after the commencement of TB treatment. We defined active TB as an ICD-9 code in the range 010–018 on at least 3 ambulatory visits or admission, and insurance claims for at least 2 TB drugs (isoniazid, rifampicin, rifamycin, rifabutin, pyrazinamide, ethambutol, streptomycin, kanamycin, amikacin, moxifloxacin, levofloxacin, ciprofloxacin, protionamide, and cycloserine) for more than 90 days, because it takes 2 months to obtain a TB culture report in clinical practice [21], [22]. ESRD patients were defined as those who received a health card for dialysis after 90 days of dialysis.

### External Validation

The main outcome of developing long-term active TB and selection criteria for identifying patients with AKI-dialysis were validated by analysis of another prospectively collected data of the National Taiwan University Hospital Study Group on Acute Renal Failure (NSARF) [23], [24]. The critical care database was constructed prospectively for outcome assurance between January 2002 and January 2008 in one medical center (National Taiwan University Hospital in Taipei, Taiwan) and its three branch hospitals in different cities [25], [26], [27], [28], [29] .The event of TB was linked to the data from Taiwan Anti-Mycobacteria Investigation (TAMI) group, a prospective collecting data recording TB events and outcomes in Taiwan [30].

### Data analysis

Continuous variables were reported as mean ± standard deviations; discrete variables were presented as counts or percentages. All data were analyzed using R software, version 2.14.1 (Free Software Foundation, Inc., Boston, MA, USA.). Two-sided *p* values<0.05 were considered statistically significant. Analyses with Cox proportional hazards regression model and propensity score model were conducted separately within each stratum to evaluate the risk of the outcomes after adjustments for all variables. For outcomes measurements, an individual was censored at active TB identification or at the end of the measurement period.

Due to the strong correlation between ESRD and TB, we used a Cox proportional hazards model with time-dependent covariates to evaluate the impact of ESRD on the risk of active TB [31], assuming that changes in ESRD status could appear at the middle time point. The time from index hospital discharge to TB event was analyzed by fitting Cox proportional hazards model. Basic model-fitting techniques for (1) variable selection, (2) goodness-of-fit assessment, and (3) regression diagnostics (including residual analysis, influence analysis, and check of multi-collinearity) were used in regression analyses to ensure good quality of results.

In specificity testing, we constructed a propensity score using a non-parsimonious multi-variable logistic regression model in an attempt to make an unbiased estimate of the confounders predicting dialysis during index admission, a binary dependent variable, under a set of covariates (Table S1 in File S1). The predicted probability derived from the logistic equation was used as the propensity score for each individual. To restore the interval validity of subsequent group comparisons and reduce the influence of observations on the overall results [32], we also truncated the samples by discarding cases in the region of non-overlap that appears with weight-trimmed propensity scores estimated by logistic regression [33], [34]. The degree of trimming was chosen to minimize mean squared error.

## Results

### Demographic characteristics of patients

We identified 2,909 patients (men, 49.9%, mean age = 61.9±14.9 years), more than 18 years of age, who had a first-time diagnosis of AKI requiring dialysis and who survived for >90 days after index discharge (AKI: dialysis group). Each AKI–dialysis patient was matched to 4 hospital patients without AKI or dialysis, for a control group of 1,1636 patients (non-AKI group: men, 45.7%; mean age = 46.2±18.4 years) (**Table 1**). Among the AKI–dialysis group, 686 recovered and no longer required dialysis after hospital discharge (recovery subgroup) (**Figure 1**). The average age of the overall cohort was 49.3±18.8 years, and the Charlson score before admission was 0.95±1.63. The prevalence of preadmission baseline and index hospital comorbidities was higher in the AKI group than in the non-AKI group, except for tumor metastasis (**Table 1**). Compared to the nonrecovery sub-group, the recovery sub-group had lower Charlson score; higher prevalences of myocardial infarction, dementia, and tumor with metastasis; and lower prevalence of chronic kidney disease.

**Table 1:** Characteristics of enrolled patients.

				AKI dialysis group (n = 2909)			
Items	Non-AKI group (n = 11636)	AKI-dialysis group (n = 2909)	*p*	*Non-recovery (n = 2223)*	*Recovery (n = 686)*	*p* ¶	*p* §
**Male**	5319(45.7%)	1452(49.9%)	<0.001	1062(47.8%)	390(56.9%)	<0.001	<0.001
**Age (years)**	46.2±18.4	61.9±14.9	<0.001	61.4±14.4	63.6±16.4	<0.001	<0.001
**Comorbidity**			<0.001				
**Charlson score**	0.43±1.03	3.01±1.92	<0.001	3.2±1.82	2.39±2.1	<0.001	<0.001
**Myocardial infarction**	49(0.4%)	55(1.9%)	<0.001	34(1.5%)	21(3.1%)	0.015	<0.001
**Congestive heart failure**	135(1.2%)	414(14.2%)	<0.001	312(14%)	102(14.9%)	0.574	<0.001
**Peripheral vascular disease**	45(0.4%)	40(1.4%)	<0.001	32(1.4%)	8(1.2%)	0.709	0.009
**Cerebrovascular disease**	264(2.3%)	254(8.7%)	<0.001	193(8.7%)	61(8.9%)	0.877	<0.001
**Dementia**	58(0.5%)	61(2.1%)	<0.001	34(1.5%)	27(3.9%)	<0.001	<0.001
**COPD**	512(4.4%)	254(8.7%)	<0.001	192(8.6%)	62(9%)	0.757	<0.001
**Rheumatologic disease**	61(0.5%)	38(1.3%)	0.001	28(1.3%)	10(1.5%)	0.701	0.006
**Peptic Ulcer**	671(5.8%)	448(15.4%)	<0.001	340(15.3%)	108(15.7%)	0.763	<0.001
**Hemiplegia**	32(0.3%)	36(1.2%)	<0.001	27(1.2%)	9(1.3%)	0.844	<0.001
**Chronic Kidney disease**	236(2%)	1754(60.3%)	<0.001	1563(70.3%)	208(30.3%)	<0.001	<0.001
**Solid tumor**	328(2.8%)	150(5.2%)	<0.001	107(4.8%)	43(6.3%)	0.139	<0.001
**Tumor with metastasis**	90(0.8%)	31(1.1%)	0.137	16(0.7%)	15(2.2%)	0.002	0.001
**Diabetes Mellitus**	841(7.2%)	1347(46.3%)	<0.001	1056(47.5%)	291(42.4%)	0.020	<0.001
**Moderate or Severe liver disease**	376(3.2%)	169(5.8%)	<0.001	127(5.7%)	42(6.1%)	0.709	<0.001
**Index hospital co-morbidities**							
**Cardiovascular**	41(0.4%)	81(2.8%)	<0.001	28(1.3%)	53(7.7%)	<0.001	<0.001
**Respiratory**	71(0.6%)	275(9.5%)	<0.001	131(5.9%)	144(21%)	<0.001	<0.001
**Hepatic**	65(0.6%)	35(1.2%)	0.009	20(0.9%)	15(2.2%)	0.014	<0.001
**Neurologic**	10(0.1%)	48(1.7%)	<0.001	36(1.6%)	12(1.7%)	0.864	<0.001
**Hematologic**	39(0.3%)	29(1%)	<0.001	25(1.1%)	4(0.6%)	0.274	0.301
**Metabolic**	3(0%)	96(3.3%)	<0.001	65(2.9%)	31(4.5%)	0.050	<0.001
**Operative categories**							
**Cardiothoracic**	51(0.4%)	49(1.7%)	<0.001	19(0.9%)	30(4.4%)	<0.001	<0.001
**Upper GI**	44(0.4%)	11(0.4%)	0.999	3(0.1%)	8(1.2%)	0.001	0.008
**Lower GI**	83(0.7%)	20(0.7%)	0.999	10(0.4%)	10(1.5%)	0.013	0.038
**Hepatobiliary**	181(1.6%)	13(0.4%)	<0.001	4(0.2%)	9(1.3%)	0.001	0.750
**Mechanical ventilation**	255(2.2%)	508(17.5%)	<0.001	253(11.4%)	255(37.2%)	<0.001	<0.001
**ICU admission during index hospitalization**	539(4.6%)	920(31.6%)	<0.001	526(23.7%)	394(57.4%)	<0.001	<0.001
**Long term outcomes**							
**ESRD**	21(0.2%)	1626(55.9%)	<0.001	1500(67.5%)	126(18.4%)	<0.001	<0.001
**TB**	53(0.5%)	66(2.3%)	<0.001	53(2.4%)	13(1.9%)	0.557	<0.001

¶ recovery subgroup *vs.* nonrecovery subgroup.

§ recovery subgroup *vs.* non-AKI group.

Abbreviations: AKI, acute kidney injury; COPD, chronic obstructive pulmonary disease; ESRD, end stage renal disease; GI, Gastrointestinal; ICU, intensive care unit; TB, tuberculosis.

### Long-term active TB (Fig. 2)

**Figure 2:**
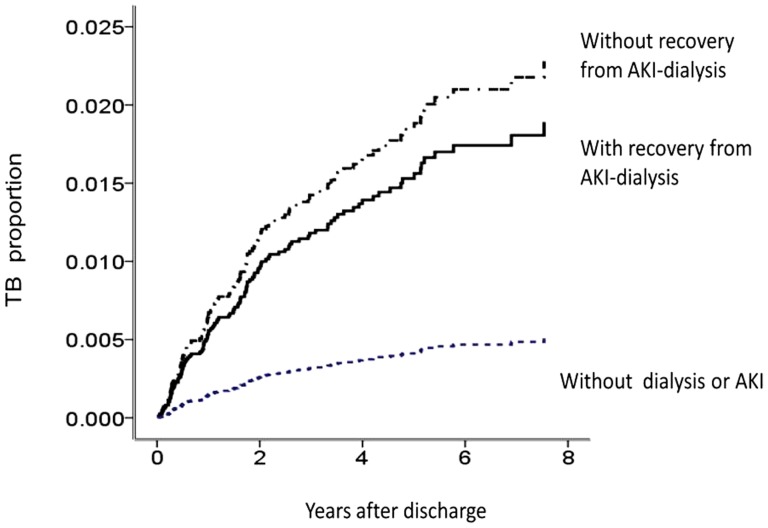
Cox proportional hazards model for long-term active TB events among the non-AKI group, recovery AKI subgroup, and nonrecovery AKI sub-group (AKI, acute kidney injury).

During a mean follow-up period of 3.6 years, a significantly greater proportion of AKI patients developed end-stage renal disease (ESRD), compared to the non-AKI group (55.9% vs. 0.2%, *p*<0.001) (**Table 1**). AKI patients had a higher long-term risk of active TB (2.3% vs. 0.5%, *p*<0.001).

The incidence of TB was 590, 650, and 83 per 100,000 person-years in the recovery sub-group, nonrecovery sub-group, and non-AKI group, respectively. The risk of TB infection in AKI–dialysis patients relative to the general population was 7.71. The hazard ratio for developing active TB, relative to non-AKI patients, was 6.39 (95% CI, 3.57–11.45; *p*<0.001) for the nonrecovery sub-group and 3.84 (95% CI, 2.07–7.10; *p*<0.001) for the recovery sub-group. Other risk factors predicting the development of active TB were age >45 years (HR, 3.64; 95% CI, 2.07–6.39; *p*<0.001), male sex (HR, 1.94; 95% CI, 1.33–2.83; *p*<0.001), and chronic obstructive pulmonary disease (HR, 1.87; 95% CI, 1.10–3.19; *p* = 0.021). This model had good validity (C-index = 0.79). However, ESRD after AKI, the time-dependent explanatory variable, was not associated with development of active TB according to our Cox proportional hazards model (HR, 1.19; *p* = 0.58). Additionally, in the AKI–dialysis group, active TB was associated with increased all-cause mortality (HR, 1.34; 95% CI, 1.03–1.74; *p* = 0.032).

**Figure 3:**
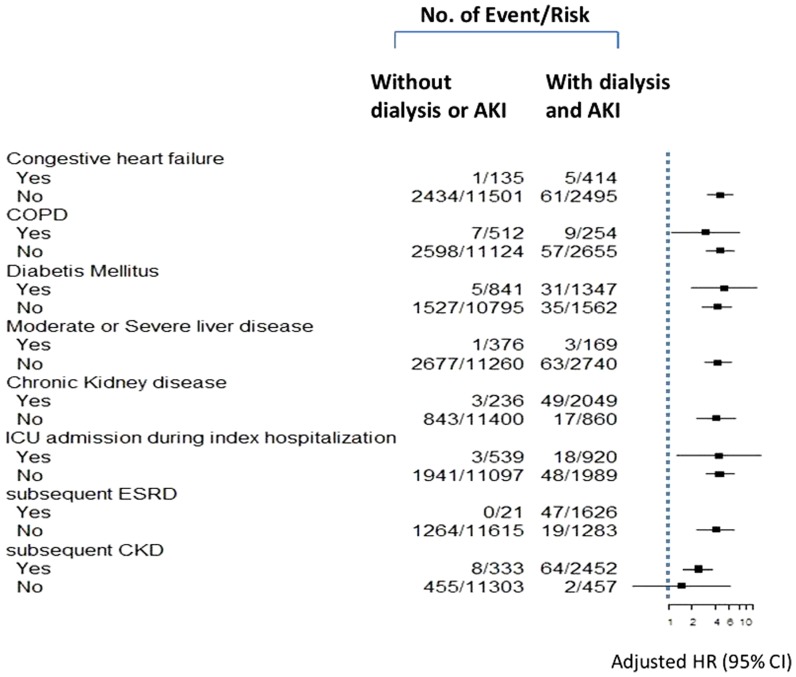
The hazard ratios (HRs) and 95% CIs for long-term tuberculosis, adjusted for the AKI–dialysis and non-AKI groups. *adjusted for age and sex. **Abbreviations**; COPD, chronic obstructive pulmonary disease; ICU.

Sensitivity analysis was consistent with our main finding that the AKI–dialysis group had a higher incidence of active TB than the non-AKI group (**Fig. 3**). This was also true for patients with or without diabetes (all, *p*<0.005). AKI-recovery groups had higher ratio of TB than non-AKI group in spite of subsequent CKD.

### External Validation using NSARF data

We validated our main findings using prospective critical care data from the multi-centers , NSARF, to guide us in conducting more broad-based research on AKI from nationally representative sources. Among 234 AKI-dialysis patients survival to discharge, 180 (76.9%) recovered from dialysis within 90 days after discharge from index admission. In the NSARF cohort, 8788 patients without AKI and survival to 90 days after hospital discharge were enrolled as controls. There were 70 (0.8%), 5 (2.8%) and 2 (3.7%) in non-AKI, recovery and non-recovery groups respectively had active TB after a mean follow-up of 3.3 years. (1.01–5.13 years). Consistent with our previous findings, the results obtained using the Cox proportional hazard model showed that recovery group (HR = 3.03; 95% CI, 1.20 −7.67; p = 0.019) and nonrecovery subgroup (HR = 4.37; 95% CI, 1.00 −19.07; p = 0.049) had high risk of developing long-term active tuberculosis compared with non-AKI.

### Specificity test using propensity matching

The risk factors predicting dialysis during index hospitalization were components of propensity scores and are listed in Table S1 **in** File S1. The propensity score for predicting dialysis during index hospitalization in all study groups was highly discriminative (estimated area under the receiver operating characteristic curve [eAUC–ROC] = 0.975), and it fit the observed binary data well (adjusted generalized *R* ^2^ = 0.819; Hosmer–Lemeshow goodness-of-fit [GOF] test, *p*<0.001).

To restore the interval validity of subsequent group comparisons and reduce the influence of observations on the overall results, we weight-trimmed 888 patients by estimated propensity scores. After careful matching, there were 526 patients in the recovery sub-group, 1,935 in the nonrecovery sub-group, and 11.196 in the non-AKI group.

Consistent with our previous finding, the AKI–dialysis group had a higher long-term incidence of active TB than the non-AKI group, after adjustment by weight-trimmed propensity scores (HR, 4.77; 95% CI, 2.09–10.89; *p*<0.001) (C-index = 0.79) (Table S2 and **Figure S1** in File S1).

We also weight-trimmed between the recovery sub-group and the non-AKI group, according to each patient's propensity score. This propensity model had good validity (eAUC–ROC = 0.984; adjusted generalized *R* ^2^ = 0.79; GOF test, *p*<0.001). After adjustment according to weight-trimmed propensity scores, patients in the nonrecovery sub-group had a greater long-term risk of active TB (HR, 1.25; 95% CI, 1.06–1.48; *p* = 0.008) (**Figure S1** in File S1) than the non-AKI group.

## Discussion

Despite recovery from AKI requiring dialysis, the most severe form of AKI, patients still had a greater long-term risk of active TB than those without AKI or dialysis during hospitalization. In addition, AKI requiring dialysis appears to be a major risk factor (7.71-increased relative risk) for active TB, independent of possible confounders. These findings are noteworthy from the perspective of a clinician providing long-term care to an individual with AKI requiring dialysis. Our study is the first to elucidate nationwide epidemiological characteristics of de novo AKI requiring dialysis in hospitalized patients and to compare the long-term risk of active TB among those continuing and not continuing dialysis and those without AKI or dialysis. The result is true in our native critical cohort; that is, patients survival from dialysis -requiring AKI, even recovery from dialysis, had higher risk of developing active TB than patients without AKI or dialysis.

Taiwan has an intermediate burden of TB. The annual incidence of TB in Taiwanese dialysis patients was 490 per 100,000 in 1997, which was 6.9 times greater than that of the general population [35]. The incidence of TB was 590 per 100,000 person-years in survivors with temporary dialysis. An epidemiological study in Taiwan found that the incidence of infection was 62–75 per 100,000 person-years during 2002–2008 [36]. The incidence in our matched patients without AKI or dialysis, 83 per 100,000 person-years, was slightly greater than the incidence in the general population. The discrepancy could be related to the greater rate of comorbidities in our study subjects, all of whom had been hospitalized.

The kidneys are uniquely positioned to serve as immunomodulatory organs [37]. There is evidence to suggest that the deleterious effects of AKI on lung function could, at least in part, be due to loss of the normal balance of immune, inflammatory, and mediators that occurs with injury of the tubular epithelium [38]. It is also notable that leukocytes passing through the kidney are exposed to a uniquely hostile environment. The kidney could well modulate leukocyte trafficking in a number of important organs,—such as the lungs,—via both adhesion molecule expression and physical characteristics of neutrophils, such as cytoplasmic “stiffness” [37], [39]. Consequently, it is reasonable to suppose that the immune system of AKI patients (mainly T lymphocytes and antigen-presenting cells) undergo disturbances that increase susceptibility to TB infection.

In a large cohort study of the risk of active TB, the hazard ratio for patients with chronic obstructive pulmonary disease (COPD) was 3 times greater than that of the general population [40]. As in patients with advanced CKD [21], COPD is a risk factor for active TB after hospital discharge. It is biologically plausible that COPD increases the risk of TB and other pulmonary infections, because hypersecretion of mucus and ciliary dysfunction can lead to impaired clearance of pathogens [41]. In agreement with a previous study, our results showed that diabetes did not affect the long-term risk of active TB in patients with advanced CKD [21]. AKI survivors with complete recovery of renal function remain at elevated risk of developing de-novo CKD, which may influence long-term survival; however, recovery of kidney function after AKI is associated with better long-term survival and renal function.^26^ While the time-dependent ESRD episode after AKI was neither related to long-term TB disease, the strong impacts of AKI-dialysis attenuated other's impacts on incident TB, ex. DM, ESRD.

TB patients with greater frailty were expected to have higher mortality and higher adjusted HRs for death, compared with non-TB patients, especially patients who recovered from AKI requiring dialysis. This phenomenon underlines the importance of evaluating the inherent frailty of research study subjects when comparing outcomes in patients with and without TB. Nonetheless, in all scenarios, patients who recovered from AKI still fared more poorly than patients without AKI or dialysis. Further studies are needed to disentangle the underlying factors contributing to this finding.

### Limitations

Major strengths of our study are the large sample and the nature of the cohort, which was based on the general population. AKI–dialysis cases in this study were presumably more severe by reason of the stringent definition that was set to avoid miss-classification of patients. Because of the retrospective nature of the claims data, the standard procedure for diagnosing TB infection, for example, acid-fast staining and culturing, was not identified. Additionally, some data had to be inferred indirectly from administrative records, including smoking status and nutritional status, because they were not available from the National Health Research Institute (NHRI). Nonetheless, we do the external data validation using the well designed, prospective collecting, native critical data, and the results were consisted with the finding from nationwide data. Some future perspectives in AKI research are highlighted with respect to novel therapeutic strategies in the prevention and control of TB.

## Conclusion

Our findings suggest that the occurrence of TB could be associated with AKI episode. New AKI therapeutic and caring strategies are needed for improved outcomes in AKI patients, targeting not only renal dysfunction alone, but also long-term frailty, which is expected to be associated with greater risk of active TB. Accordingly, the high incidence of TB after recovery from AKI requiring dialysis will correspond to high mortality. Our results raise concerns that the increasing global burden of AKI will include increased incidence of active TB.

## Supporting Information

File S1 **Table S1.** Demographics and comorbidities added into a non-parsimonious propensity model to predict dialysis during index hospitalization in the AKI-dialysis and non-AKI groups. **Table S2.** Characteristics of patients in the AKI-dialysis and non-AKI groups at index hospitalization matched by weight trimmed logistic regression estimated propensity scores. **Figure S1.** Cox proportional hazards model for long-term active TB events, stratified by AKI–dialysis status at index hospitalization and adjusted by weight-trimmed logistic regression estimated propensity scores.
(DOC)
