# Supplementary material for: Increased Risk of Active Tuberculosis following Acute Kidney Injury: A Nationwide, Population-Based Study
Source: PLoS One. 2013 Jul 25;8(7):e69556. doi: 10.1371/journal.pone.0069556 (PMC3723893; doi:10.1371/journal.pone.0069556)

**Supporting Information**

Table S1. Demographics and comorbidities added into a non-parsimonious propensity model to predict dialysis during index hospitalization in the AKI-dialysis and non-AKI groups

| **Items** | **OR** | **lower 95% CI** | **upper 95% CI** | ***P*** |
| --- | --- | --- | --- | --- |
| **Age > 40** | 2.25 | 1.82 | 2.80 | <0.001 |
| ***Baseline comorbidities*** |  |  |  |  |
| **Charlson score** | 1.95 | 1.65 | 2.28 | <0.001 |
| **Chronic Kidney disease** | 37.65 | 26.11 | 54.95 | <0.001 |
| **Moderate or Severe liver disease** | 0.19 | 0.12 | 0.31 | <0.001 |
| **COPD** | 0.26 | 0.18 | 0.39 | <0.001 |
| **Solid tumor** | 0.01 | 0.00 | 0.02 | <0.001 |
| **Tumor with metastasis** | 0.23 | 0.13 | 0.39 | <0.001 |
| **Myocardial infarction** | 0.20 | 0.09 | 0.44 | <0.001 |
| **Peptic Ulcer** | 0.50 | 0.35 | 0.70 | <0.001 |
| **Hemiplegia** | 0.21 | 0.07 | 0.66 | 0.008 |
| **Cerebrovascular disease** | 0.68 | 0.44 | 1.05 | 0.088 |
| **Diabetes Mellitus** | 1.35 | 0.98 | 1.87 | 0.067 |
| ***Index hospital comorbidities*** |  |  |  |  |
| **Hematologic** | 2.66 | 0.93 | 7.11 | 0.062 |
| **Metabolic** | 111.39 | 32.94 | 514.79 | <0.001 |
| **Respiratory** | 3.70 | 2.27 | 6.01 | <0.001 |
| **Neurologic** | 4.52 | 1.49 | 15.66 | 0.011 |
| **ICU admission during index hospitalization** | 7.31 | 5.44 | 9.76 | <0.001 |
| **Mechanical ventilation** | 1.66 | 1.12 | 2.45 | 0.011 |
| ***Operative categories*** |  |  |  |  |
| **Hepatobiliary** | 0.18 | 0.04 | 0.59 | 0.142 |

Adjusted generalized R^2^ =0.819, Hosmer-Lemeshow goodness of fit [GOF] test P <0.001

**Abbreviations,**

AKI, acute kidney injury; CI, confidence interval; COPD, chronic obstructive pulmonary disease; ESRD, end stage renal disease; GI, Gastrointestinal; NSAID, nonsteroidal anti-inflammatory agents/analgesics.

Table S2, Characteristics of patients in the AKI-dialysis and non-AKI groups at index hospitalization matched by weight trimmed logistic regression estimated propensity scores.

|  |  | |  |  |
| --- | --- | --- | --- | --- |
| **Items** | **Non-AKI group (n=11196)** | | **AKI-dialysis group (n=2461)** | ***p*** |
| **Male** | 5087(45.4%) | | 1245(50.6%) | <0.001 |
| **Age** | 46.0±18.4 | | 61.3±15.1 | <0.001 |
| **Comorbidity** | |  |  | <0.001 |
| **Charlson score** | 0.39±1.0 | | 2.82±1.9 | <0.001 |
| **Myocardial infarction** | 44(0.4%) | | 44(1.8%) | <0.001 |
| **Congestive heart failure** | 132(1.2%) | | 297(12.1%) | <0.001 |
| **Peripheral vascular disease** | 45(0.4%) | | 34(1.4%) | <0.001 |
| **Cerebrovascular disease** | 261(2.3%) | | 197(8%) | <0.001 |
| **Dementia** | 58(0.5%) | | 41(1.7%) | <0.001 |
| **COPD** | 418(3.7%) | | 203(8.2%) | <0.001 |
| **Rheumatologic disease** | 60(0.5%) | | 29(1.2%) | 0.001 |
| **Peptic Ulcer** | 596(5.3%) | | 356(14.5%) | <0.001 |
| **Hemiplegia** | 30(0.3%) | | 28(1.1%) | <0.001 |
| **Chronic Kidney disease** | 236(2.1%) | | 1649(67%) | <0.001 |
| **Solid tumor** | 269(2.4%) | | 121(4.9%) | <0.001 |
| **Tumor with metastasis** | 64(0.6%) | | 27(1.1%) | 0.006 |
| **Diabetes Mellitus** | 837(7.5%) | | 1056(42.9%) | <0.001 |
| **Moderate or Severe liver disease** | 194(1.7%) | | 137(5.6%) | <0.001 |
| **Index hospital co-morbidities** |  | |  | <0.001 |
| **Cardiovascular** | | 40(0.4%) | 65(2.6%) | <0.001 |
| **Respiratory** | 71(0.6%) | | 202(8.2%) | <0.001 |
| **Hepatic** | 4(0%) | | 459(18.7%) | 0.009 |
| **Neurologic** | 59(0.5%) | | 25(1%) | <0.001 |
| **Hematologic** | 10(0.1%) | | 40(1.6%) | <0.001 |
| **Metabolic** | 39(0.3%) | | 24(1%) | <0.001 |
| **Operative categories** |  | |  | <0.001 |
| **Cardiothoracic** | | 51(0.5%) | 43(1.7%) | <0.001 |
| **Upper GI** | 39(0.3%) | | 9(0.4%) | 0.851 |
| **Lower GI** | 82(0.7%) | | 16(0.7%) | 0.792 |
| **Hepatobiliary** | 36(0.3%) | | 10(0.4%) | <0.001 |
| **Mechanical ventilation** | 253(2.3%) | | 396(16.1%) | <0.001 |
| **ICU admission during index hospitalization** | 538(4.8%) | | 720(29.3%) | <0.001 |
| **mortality** | 949(8.5%) | | 1081(43.9%) | <0.001 |
| **ESRD** | 21(0.2%) | | 1412(57.4%) | <0.001 |
| **TB** | 51(0.5%) | | 56(2.3%) | <0.001 |

**Abbreviations,**

AKI, acute kidney injury; COPD, chronic obstructive pulmonary disease; ESRD, end stage renal disease; GI, Gastrointestinal; ICU, intensive care unit; NSAID, nonsteroidal anti-inflammatory agents/analgesics; TB, tuberculosis.

**Figure S1**

Cox proportional hazards model for long-term active TB events, stratified by AKI–dialysis status at index hospitalization and adjusted by weight-trimmed logistic regression estimated propensity scores.


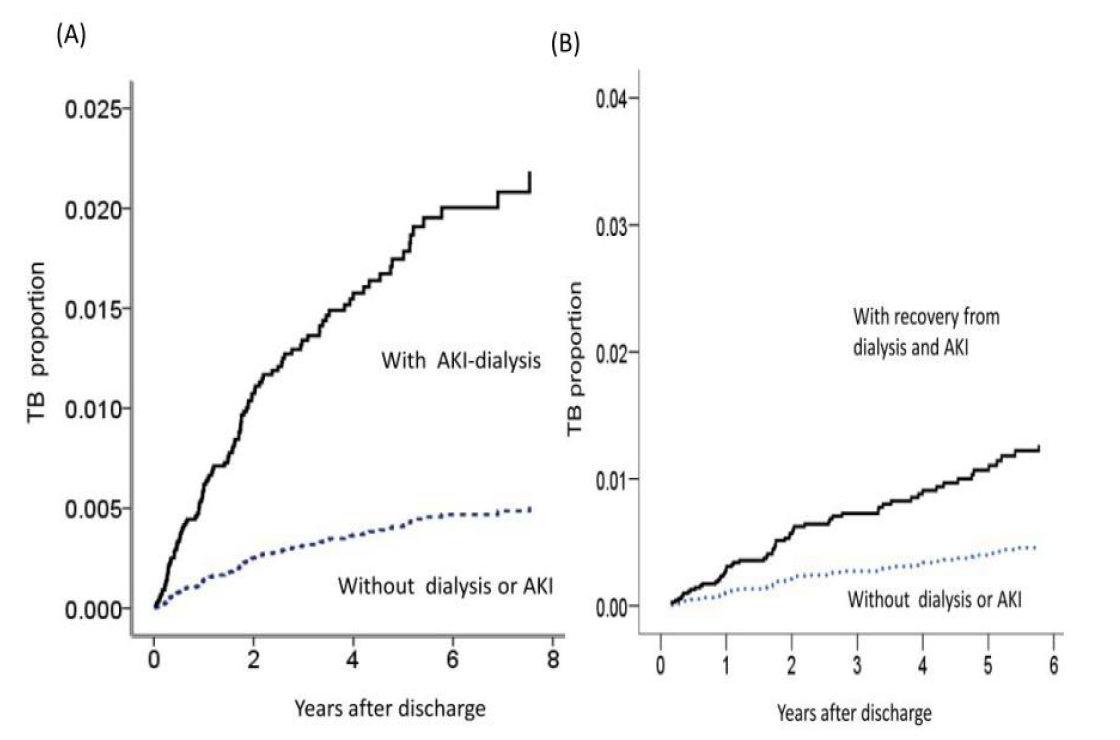

Supplement: File S1 — Table S1. Demographics and comorbidities added into a non-parsimonious propensity model to predict dialysis during index hospitalization in the AKI-dialysis and non-AKI groups. Table S2. Characteristics of patients in the AKI-dialysis and non-AKI groups at index hospitalization matched by weight trimmed logistic regression estimated propensity scores. Figure S1. Cox proportional hazards model for long-term active TB events, stratified by AKI–dialysis status at index hospitalization and adjusted by weight-trimmed logistic regression estimated propensity scores. (DOC) [file pone.0069556.s001.doc]
